# Social and behavioral correlates of sleep health among adults receiving medication treatment for opioid use disorder

**DOI:** 10.1080/16066359.2025.2530997

**Published:** 2025-08-06

**Authors:** Lois S. Sadler, Sangchoon Jeon, Ahmad Ibrahim, Declan Barry, Uzoji Nwananji-Enwerem, Dustin Scheinost, Henry Yaggi, Nancy S. Redeker

**Affiliations:** a Yale University School of Nursing, Orange, CT, USA;; b Yale School of Medicine, New Haven, CT, USA;; c Department of Psychiatry and Child Study Center, Yale School of Medicine, New Haven, CT, USA;; d Clinical Epidemiology Research Center VA CT Healthcare Center, West Haven, CT, USA;; e University of Connecticut School of Nursing, Storrs, CT, USA

**Keywords:** Opioid use disorder, medications for opioid use disorder, sleep health, social determinants of health

## Abstract

**Objectives:**

Opioid use disorder (OUD) and its treatment (MOUD) are associated with altered sleep health. The purposes are to (1) describe profiles of sleep health among adults using medication for opioid use disorder (MOUD) and (2) examine the associations between multi-level individual, family, neighborhood, and social characteristics and sleep profiles. We hypothesized that poor quality of life, adverse life experiences, addiction behavior, dysfunctional family and social interactions, and negative neighborhood characteristics are associated with negative profiles of sleep health.

**Methods:**

This study comprised baseline analyses of the NIH/HEAL-funded CLOUDS study (Collaboration Linking Opioid Use Disorder and Sleep). We obtained self-report measures of sleep health and indicators of multi-level individual, family, and neighborhood factors. We identified sleep health profiles with K-means cluster analysis and examined the associations between these multi-level factors and sleep health profiles.

**Results:**

The sample included 165 participants (M age = 42, SD =11.4 years; *N* = 73/42.2% female; *N* = 37/22.4% Black or more than one race). We identified four sleep health profiles: Healthy sleep (Profile A; 30.3%); mild insomnia/late sleep timing (Profile B: 20%); clinical insomnia/long sleep (Profile C: 25.5%); and insomnia with excessive daytime sleepiness (Profile D: 23.6%). There were statistically significant differences across sleep profiles in physical and psychological health, addiction use and risk, family function, neighborhood esthetic quality, and perceptions of community support, with more adverse factors associated with poorer sleep health.

**Conclusions:**

Research is needed to understand the causal directions of these relationships and promote multi-level interventions to promote sleep health.

## Introduction

Opioid use disorder (OUD) afflicts about 2.5 million people in the United States (Jones et al. 2023) and is a pressing medical, economic, and societal concern. Medication for opioid use disorder (MOUD) with methadone or buprenorphine is a first-line treatment for this disorder. Sleep problems are common among people with OUD (Hernandez and Griggs 2024). They may be important determinants of additional adverse health outcomes, including lack of treatment adherence (Wilkerson and McRae-Clark 2021). Despite the benefits of MOUD, it is also associated with impairments in sleep (Chrobok et al. 2020; Berro et al. 2022; Finlay et al. 2023; Hernandez and Griggs 2024), Persons on MOUD may continue to have poorer sleep than adults in the community who do not have OUD. Sleep difficulties manifest in reports of poor sleep quality and insomnia symptoms, central and obstructive sleep apnea, increased stage N1 sleep, increased sleep latency, and irregular sleep cycles (Finlay et al. 2023). Despite increased attention to sleep characteristics, most studies addressed isolated aspects of sleep deficiency but have not evaluated the multi-dimensional elements of sleep from the perspective of sleep health (Buysse 2014). A better understanding of sleep profiles that reflect the multi-dimensional nature of sleep health may assist in defining more precise targets for sleep-promoting intervention than isolated characteristics (e.g. sleep duration) alone.

Adverse individual, family/social, and economic factors (social determinants of health) and stigma are common and may contribute to the development and perpetuation of OUD (Jalali et al. 2020a, 2020b; Cambron et al. 2023; McCurry et al. 2023; Nwanaji-Enwerem et al. 2024; Nwanaji-Enwerem et al. 2024). They may also influence access and retention on MOUD (Nwanaji-Enwerem et al. 2024). Social determinants are also associated with sleep health (Grandner et al. 2015; Jean-Louis et al. 2022; Kim et al. 2023; Nwanaji-Enwerem et al. 2024). However, few studies have considered the associations between individual, family/social, and environmental factors and multi-dimensional profiles of sleep health among people with OUD, including those on MOUD.

**Figure 1.**
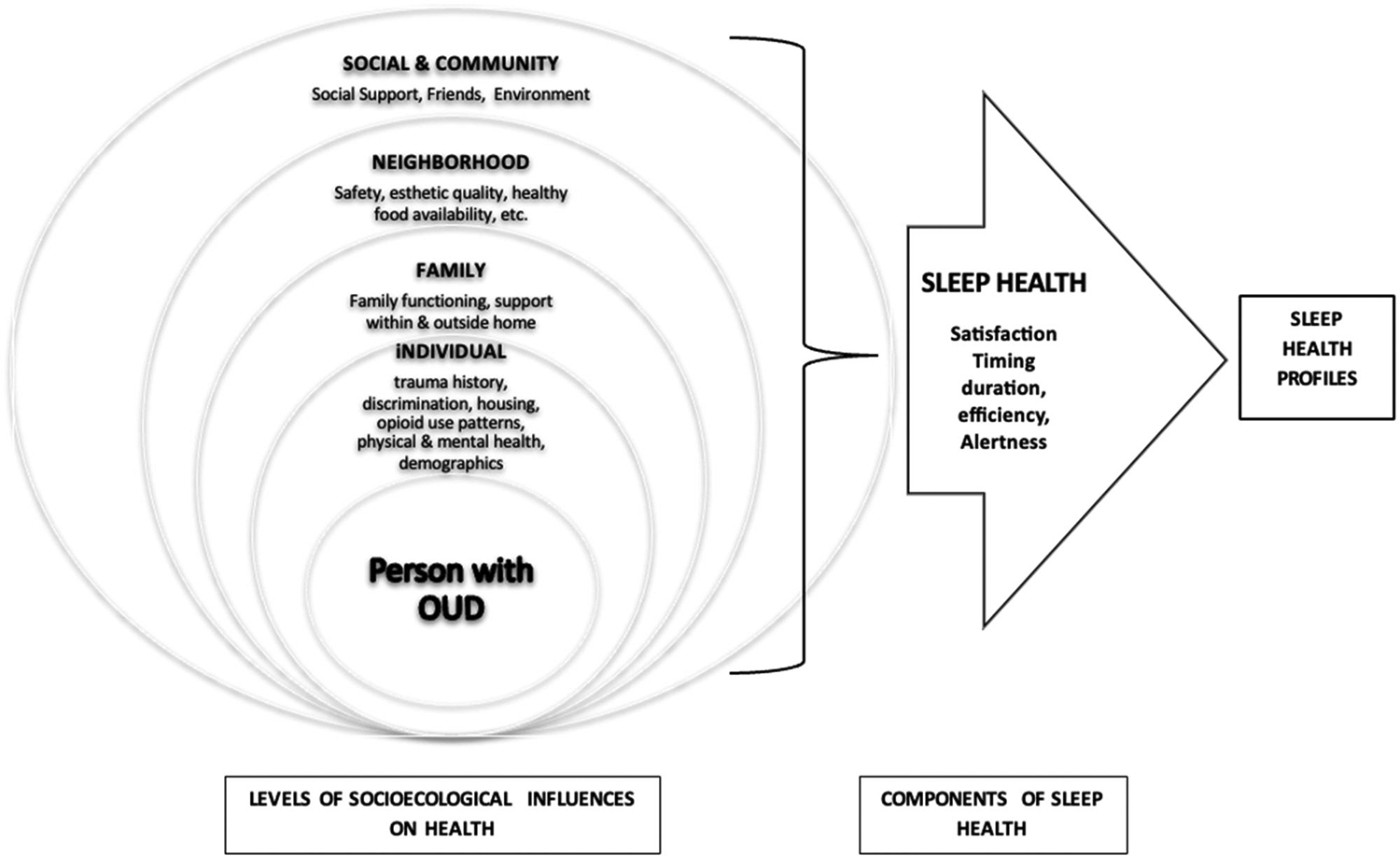
Socioecological variables and sleep health.

The Social Ecological Model (Bronfenbrenner 1979) and the National Institutes of Health/National Institute on Minority Health and Health Disparities (2017) posit that multi-level individual/behavioral, family, and social/environmental factors contribute to health, such that proximal level factors usually have more significant influence on health outcomes than distal level factors (Bronfenbrenner 1979; Rosa and Tudge 2013; Common Fund, 2017; Jalali et al. 2020a, 2020b). Our study was guided by a conceptual model (Figure 1) reflecting a multi-level ecological model, which has been documented to influence outcomes for those with OUD. These levels represent protective and risk variables (Jalali et al. 2020a; Lin et al. 2024). Application of the model to sleep health among individuals with OUD provides a more nuanced understanding of behavioral, family/social, and environmental strategies associated with promoting sleep health than models that only examine clinical and demographic correlates.

The purposes of this study were to (1) describe profiles of sleep health among adults with OUD who were on MOUD and (2) examine the associations between multi-level individual, family/social, and neighborhood characteristics and sleep profiles among these individuals. We hypothesized that poor quality of life, adverse life experiences, addiction behavior (e.g. recurring use of opioids, polydrug use, or continued exposure to substance-using peers), dysfunctional family and social interactions, and negative neighborhood characteristics are associated with more negative profiles of sleep health.

## Methods

This report is a sub-study of the “CLOUDS” observational cohort study funded by the NIH HEAL initiative (U01HL150596). The CLOUDS study was designed to understand the contributions of sleep to relapse and retention on MOUD, and participants were followed for six months. In the current paper, we report baseline data. The Institutional Review Board approved the study, and all participants provided written informed consent.

Participants were recruited from a not-for-profit community-based open-access opioid treatment center in an urban area in the Northeastern United States that was affiliated with a major university. We included participants with OUD who sought MOUD treatment at this center. Participants were 18 years or older, English-speaking, met current DSM-5 criteria for OUD, were eligible for methadone or buprenorphine treatment, and had been using MOUD for no more than 24 weeks. This time frame was due to the primary aim of the parent study to understand the role of sleep in relapse and retention early in treatment.

Participants were excluded if they were pregnant, worked night, evening, or rotating shifts, or if they were acutely psychotic, suicidal, or homicidal, or had been given prescription opioids for acute pain, chronic pain, or palliative care. Participants with pending legal actions or planned relocation that made it unlikely that they could complete the study, and those receiving continuous positive airway treatment for sleep apnea, were excluded. We did not exclude those who had sleep-disordered breathing.

Due to the use of functional magnetic resonance imaging (fMRI) scanning in the parent study, we also excluded individuals with contraindications to fMRI including severe claustrophobia; history of brain surgery; penetrating, neurovascular, infectious, or significant brain injury vascular or hematologic disorders that might affect blood-oxygen-level-dependent signal (e.g. sickle cell disease), auditory/visual impairment that might interfere with stimuli processing, and any metal implant, hardware, or metal exposure.

We prescreened medical records to determine eligibility. Research staff members contacted participants who were prescreened. We provided a detailed explanation of the study before obtaining written informed consent. Participants provided written informed consent. They underwent a more thorough screening and were enrolled if they met the study criteria. Participants in the parent study were followed for six months. They also completed overnight polysomnography, actigraphy, magnetic resonance imaging, and biomarker collection. However, these data will be reported separately from the current paper.

## Variables and measures

Data on sleep health and individual, family, neighborhood, and social/community variables, based on the social-ecological model (See Figure 1), were obtained through interviews, medical record reviews, and questionnaires completed by participants.

### Sleep health

Based on Buysse’s Model of Sleep Health (Buysse 2014), we included self-report indicators of self-reported sleep satisfaction, timing, duration, efficiency, and alertness reflecting each model component.

We used the well-validated Pittsburgh Sleep Quality Index (PSQI) (Buysse et al. 1989) to measure self-reported sleep satisfaction, duration, and efficiency. Midsleep timing was elicited with the raw data from the sleep onset and offset times recorded in the PSQI. The PSQI scores range from 0 to 21, with higher scores indicating poorer sleep quality. A score > 5 on the PSQI indicates poor sleep quality. Scores on the sleep duration and efficiency components range from 0 to 3 (Johns 1991).

The 8-Item PROMIS Sleep Disturbance Short Form (Yu et al. 2011) was used to measure sleep satisfaction. The scores range from 8 to 40, with higher scores indicating poorer sleep. The PROMIS scales have been standardized so that the population mean is 50 and the standard deviation is 10. We used this metric (Yu et al. 2011).

The 7-item Insomnia Severity Index was also used to indicate sleep satisfaction. The total score ranges from 0 to 28. This scale can be scored from 8 to 14, indicates mild insomnia, and a score of 15 or higher indicates clinical insomnia symptoms (Bastien et al. 2001). However, a cutoff score > 10 has also been used and is sensitive and specific for clinical insomnia in community adults. (Morin et al. 2011).

The Epworth Sleepiness Scale (ESS), a well-validated 5-item measure, was used to indicate daytime alertness/sleepiness (Johns 1991). Scores range from 0 to 24, with scores over 10 indicating excessive daytime sleepiness.

### Multilevel social ecological characteristics

We selected indicator variables to reflect the levels and major concepts of the Social Ecological Model (Bronfenbrenner 1979) and the NIMHD Framework (2017), as designated in the study’s conceptual model and the literature on substance use and sleep. These include individual, family, neighborhood, and social/community factors.

#### Individual-level factors.

We selected individual-level variables as those most proximal to the individual and consistent with the extant literature relevant to sleep and OUD. We included clinical and demographic characteristics (gender, race, income, ethnicity, others in the household, level of education, and employment) obtained through participant interviews, medical records data, and the Charlson comorbidity index (Charlson et al. 1987). We also elicited opioid use, addiction, health-related quality of life, history of childhood trauma, stigma, and discrimination *via* self-reports.

Self-reported illicit opioid use was measured using the Timeline Follow-back method, a reliable and valid calendar-based method (Robinson et al. 2014; Sobell, 1988). Opioid use was confirmed with urine drug testing.

The Brief Addiction Monitor is a 17-item measure of progress among persons in treatment for substance use disorders (Cacciola et al. 2013). It contains three subscales: risk factors (triggers for use with a cut-point of ≥12), protective factors (facilitators or supports for nonuse with a cut-point of ≤ 12), and use score (substance use with a clinical cut-point of ≥ 1).

We used the World Health Organization Quality of Life instrument (WHOQOL-BREF) (Skevington et al. 2004). The WHO developed this 26-item Likert measure to assess four quality of life domains: physical health, psychological health, social relationships, and environment. Higher scores on each domain are associated with better quality of life. The score range is 4–20 for each domain.

Childhood trauma was assessed with the Childhood Trauma Questionnaire (CTQ), a 28-item retrospective self-report questionnaire with scores ranging from 25 to 125 (Bernstein et al. 1994). Participants rated experiences during adolescence on a 5-point Likert scale, ranging from ‘never true’ to ‘very often true.’ The CTQ comprises five trauma subscales (emotional abuse, emotional neglect, physical abuse, physical neglect, and sexual abuse) rated on a scale from 5 (indicating less trauma) to 25 (indicating more trauma). Subscale scores range from 5 to 25, with higher scores indicating more trauma. The subscales are summed for a total score, which provides a general measure of childhood trauma (range 25 to 125).

The 12-item Brief Opioid Stigma Scale (BOSS) elicited distinct stereotypes associated with OUD. The scale includes three subscales, which gauge the following aspects: perceived stigma or awareness of stereotypes (‘aware’), internalized stigma or agreement with stereotypes (‘agree’), and decrease in self-esteem related to OUD (‘harm’). Subscale scores for each component range from 4 to 20, with higher scores indicating more significant stigma (Yang et al. 2019).

The Intersectional Discrimination Index (IDI) elicits perspectives of everyday discrimination arising from gender, ethnicity, and mental health diagnosis. The primary scale consists of nine Likert-type items with four response options, ranging from 1 (‘never’) to 4 (‘many times’). Scores range from 9 to 36, with higher scores indicating a higher level of experienced discrimination. (Scheim and Bauer 2019).

#### Family Level Variable.

Family functioning was assessed using the 12-item General Family Functioning Subscale of the McMaster Family Assessment Device (Mansfield et al. 2015). Individuals rate their satisfaction with family functioning. The measure has been used widely with diverse families, including those experiencing addiction (Hosseinbor et al. 2012; Hamilton and Carr 2016). Scores range from 12 to 48 with higher scores indicating less satisfaction with family functioning.

#### Neighborhood Level Variable.

The Neighborhood Environment Scale includes six subscales, on which participants rate their perceptions of the neighborhood based on esthetic quality (6 items), walking environment (10 Items), availability of healthy foods (4 items), safety (4 items, violence (4 items), and social cohesion (5 items) Subscale scores are computed by averaging Likert ratings (1 to 5) for the items of each scale. Higher scores denote more disadvantaged neighborhood environments (Mujahid et al. 2007).

#### Social and Community Level Variables.

The 24-item Community Assessment Inventory (CAI) (Brown et al. 2004; Kelly et al. 2010) was developed and tested with participants in outpatient drug treatment. It elicits perceptions of support available across the following domains: household members, family outside the home, friends, and community. Likert responses (1–4) are summed for each subscale and combined for a total score. Total scores range from 24 to 96 with higher scores indicating greater support.

The Social Network Index measures participation in social relationships, including the number of high-contact roles within 12 types of relationships (network diversity), the number of people in the individual’s social network, and the number of embedded networks (the number of network groups in which an individual is active) (Cohen et al. 1997; Pantell et al. 2013). Higher scores indicate access to more relationships and networks.

## Statistical methods

All statistical analyses were performed with SAS software, Version 9.4 (SAS Institute Inc, Cary, NC, USA). To understand the underlying latent factors of the sleep variables, we performed exploratory factor analysis (EFA) to extract the principal components with insomnia severity, daytime sleepiness, and sleep outcomes from PSQI (i.e. total sleep quality, duration, latency, efficiency, and midpoint). Principal components were determined with eigenvalues of one or greater and Oblique rotation, which allows correlations between principal components.

We used a K-means cluster approach to identify sleep health profiles with the indicators of sleep characteristics (sleep satisfaction, efficiency, duration, timing, and alertness). The number of profiles was determined based on clinical interpretability and statistical criteria (i.e. to maximize the Pseudo F statistic and Cubic Clustering Criterion). We characterized the profiles of the sleep variables with means and standard deviations and identified conceptual labels that captured these descriptions. To identify the correlates of the sleep health profiles, we compared the means of the individual/behavioral, family, neighborhood, and societal factors variables across the profiles. We used multinomial logistic regression to predict the sleep profiles with each variable. We developed a parsimonious multiple regression model with individual variables that had at least a marginal p-value of 0.1 in bivariate analysis. The model selection was determined as backward selection and confirmed using stepwise selection. We then tested whether the family, neighborhood, and social/community variables were still significant in the selected parsimonious model with the individual-level variables and demographics. The adjusted odds ratios (ORs) and 95% confidence intervals were estimated from the parsimonious models.

## Results

The demographic data are presented in Table 1, and descriptive data with Cronbach’s alpha values and score ranges are reported in Table 2. This study included 165 participants [Mean Age= 42.0 (SD = 11.4), Range= 24 – 71 years], of whom 44.2% were female; 75.2% were White; 9.7% were Black/African American; 12.7% reported more than one race; and 18.2% were Hispanic or Latino (See Table 1). Among these, 35.76% were unemployed and looking for work, and 10.9% were unhoused. Table 2 presents the descriptive statistics for the individual, family, neighborhood, and social/community level variables. Measures had good reliability with Cronbach’s alpha coefficients of 0.8 or greater, while the subscales of the Brief Addiction Monitor had alpha coefficients of 0.55 to 0.65. Participants used Fentanyl or opioids for 2.4 days per week on average [i.e. 34.5% (SD = 38.8) of 7 days].

Table 3 presents the principal components from the EFA with the sleep health variables. There were three components. The first component (‘insomnia symptoms and sleep quality’) was primarily explained by insomnia, the total PSQI score, and self-reported sleep latency and efficiency. Component 2 was characterized by sleepiness and short sleep duration, while delayed sleep midpoints characterized Component 3.

Table 4 presents the results of the K-means cluster analysis that produced four sleep profiles (Profile A: Healthy sleep; Profile B: Mild insomnia with delayed sleep timing; Profile C: Clinical insomnia with long sleep duration; and Profile D: Clinical insomnia with excessive daytime sleepiness). The means and standard deviations for each variable are presented in the table, and these descriptors were based on interpretation of these levels. Profiles C and D participants had the worst insomnia symptoms, sleep disturbance, and sleep quality scores. Participants in Profile D had the most sleepiness [Epworth Scale = 12.49 (SD = 5.86)], the most prolonged sleep latency [76.08 (SD = 74.04) minutes], the shortest sleep duration [5.38 (1.63) hours], and the lowest sleep efficiency [67.3 (SD = 19.6) %]. Participants in Profile C had the most extended sleep duration of [*M* = 8.64 (SD = 1.68)] hours but the poorest sleep efficiency of 57.6% (SD = 13.6). Those in Profile B had a short average sleep duration of 6.79 (SD = 1.59) hours and the latest sleep midpoint (5:35 AM) with moderate insomnia and other sleep health characteristics. Participants in Profile A had relatively healthy sleep (ISI < 7), sleep disturbance (PROMIS <50), normal sleep duration (7 to 8 h), and sleep efficiency (>80%).

**Figure 2.**
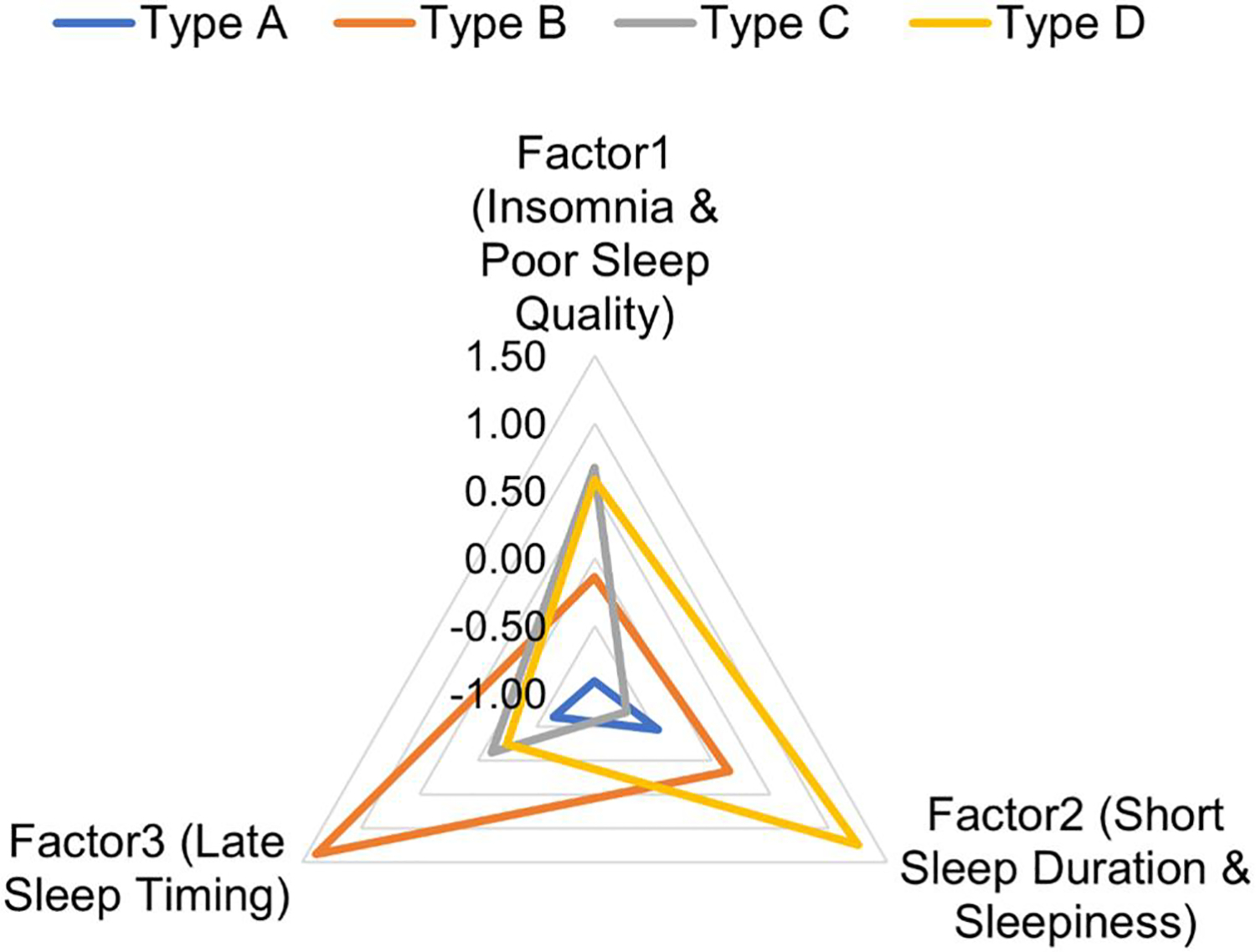
Sleep health component means by the sleep health profiles.

Figure 2 shows how the three principal components of sleep health differed across the profiles. Table 4 shows the demographic characteristics across the four profiles. The proportion of participants who lived in their own homes was highest in Profile B (91.2%) and the lowest in Profile D (64.1%). None of the other demographic characteristics were statistically different across the sleep health profiles.

Table 5 presents the means and standard deviations of the individual, family, neighborhood, and social/community level variables across the sleep health profiles. The p-values were obtained from the multinomial logistic regression without adjustment (*Unadjusted p-values) and with adjustment for (***Adjusted p-values) the variable, living in one’s own home. For the individual level variables, profiles C and D had statistically greater means on childhood trauma (Unadj. *p* = .0469/Adj. *p* = .1513) and intersectional day-to-day discrimination (Unadj. *p* = .0566/Adj. *p* = .0496). Participants in Profiles A and B had better physical (Unadj. *p*=.0001/Adj. *p* = .0002) and psychological (Unadj. *p* = .0480/Adj. *p* = .0955) health and lower scores on use of opioids and other substances on the Brief Addiction Monitor (Unadj. *p* = .0221/Adj. *p* =.0327) and risk factors (Unadj. & Adj. *p*<.0001). Profile C and D participants had significantly worse family functioning (Unadj. *p*=.0052/Adj. *p*=.0081) and neighborhood esthetic quality (Unadj. *p* = .0130/Adj. *p* = .0545). Participants in Profile A reported a better community support experience (Unadj. *p* = .0070/Adj. *p* = .0112) and environmental satisfaction (Unadj. *p* = .0277/Adj. *p*=.0693).

**Figure 3.**
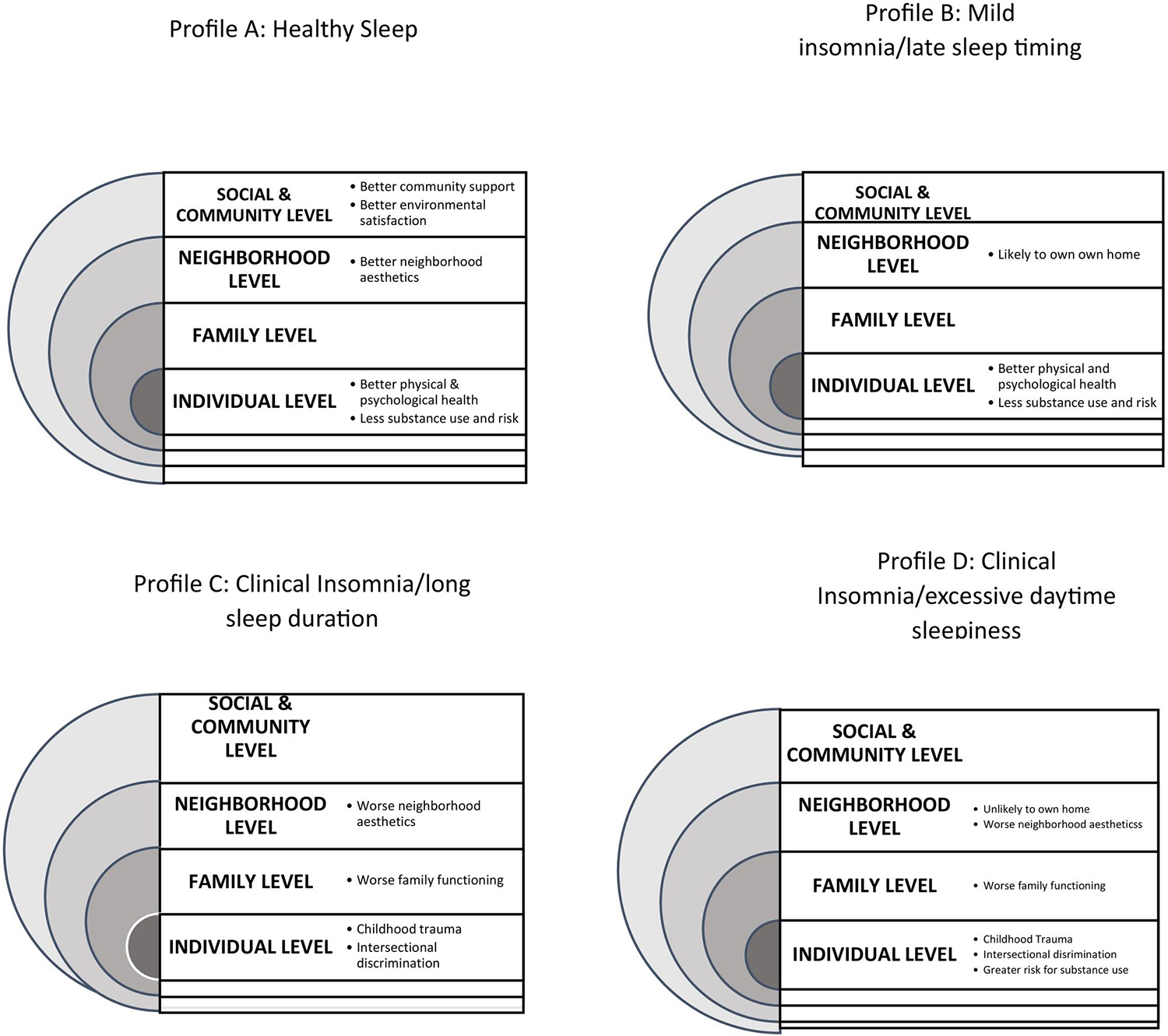
Social/community, neighborhood, and individual characteristics of people in the sleep profiles.

Table 6 shows the adjusted odds ratios (Adj. ORs) and 95% confidence intervals (CIs) for the significant correlates of the sleep health profiles in the parsimonious models with demographic and other individual characteristics. We estimated the odds ratios (ORs) and 95% confidence intervals (CIs) for sleep health profiles B, C, and D compared to profile A, representing healthy sleep. The parsimonious model included only variables with a p-value less than 0.10. After adjusting for BAM risk factors and housing status (own vs. other living situations), physical health was not significantly associated with the sleep health profiles (*p* = 0.0765). Although the confidence intervals slightly included 1, the ORs of 0.61 (Profile C vs. A) and 0.57 (Profile D vs. A) suggest lower physical health compared to those with healthy sleep. After adjusting for physical health and housing status, we found that participants with a higher BAM risk factor score were more likely to belong to profiles B (OR = 2.68), C (OR = 2.17), and D (OR = 2.85) compared to profile A (*p* = 0.0066). Additionally, the parsimonious model revealed that participants living in their own homes were more likely to belong to profile B compared to other profiles. None of the other family, neighborhood, or social/community level variables were statistically significantly different across the profiles after adjusting for the individual-level variables and living in one’s own home. However, the neighborhood esthetic quality score was lower (lower score = better quality) for (a) people living in their own homes compared to those who were not (Mean = 2.47 (1.06) vs. Mean = 3.35 (1.04) and (b) for those in Profile D (Adj. OR = 1.80, 95% CI [1.05, 3.08]) compared to Profile A after adjusting for physical health, addiction risk factors, and living in one’s own home. Figure 3 illustrates participants’ individual, family, neighborhood, community, and social characteristics in profiles A through D.

## Discussion

We identified four profiles of sleep health in participants on MOUD, advancing previous studies that examined isolated measures of sleep in people with OUD or those on MOUD (Chrobok et al. 2020; Berro et al. 2022; Finlay et al. 2023; Hernandez and Griggs 2024). Although it is often presumed that OUD is associated with poor sleep health, it is notable that almost a third of participants had overall positive aspects of self-reported sleep health, with slightly low sleep efficiency, while others had mild insomnia symptoms with late sleep timing (20%), on average clinical levels of insomnia symptoms with long sleep (25.5%) and clinical levels of insomnia symptoms with excessive daytime sleepiness (23.6%), based on Bastien et al. (2001) cutoff scores, while all but participants in profile A had some insomnia using the earlier criteria (Morin et al. 2011). Except for participants in Profile A (good sleep), all had prolonged sleep latency, indicating sleep onset insomnia. Profile B had a delayed sleep midpoint, suggesting that they had delayed sleep phases, while participants in Profile D were the only ones with significant excessive daytime sleepiness.

Our findings, based on the multi-level Social Ecological Model (Bronfenbrenner 1979) and the more recent Social-Ecological Framework of the Opioid Crisis (Jalali et al. 2020a), suggest multi-level factors that are most closely associated with sleep health among individuals who are early in treatment with MOUD. However, the findings must be interpreted cautiously due to the study’s cross-sectional nature and the post-hoc analysis, including the use of both adjusted and unadjusted analyses. Statistically significant differences and trends across the sleep health profiles at each level of the correlates (individual, family, neighborhood, and social/community) suggest the importance of multiple-level factors that may serve as risk and protective factors for sleep health among people with MOUD. Some aspects are long-standing experiences and perceptions (e.g. childhood trauma), and others may be more proximal in time to opioid use (e.g. current health, frequency of opioid use).

There were meaningful differences across the profiles in individual-level characteristics. Perceptions about how individuals perceived their own childhood and substance use histories, discrimination, family, and social/community were generally associated with membership in Profile A (good sleep health), Profile B (mild insomnia), or both. Participants with the healthiest sleep had the most positive individual characteristics and experiences relative to the other profiles. However, the relationships varied considerably based on the metrics used, and not all differences were statistically significant.

Scores on childhood trauma for all groups were over 35, suggesting significant trauma. These levels were somewhat lower than those of young women who were socially and racially marginalized, living in the same community, and who were not opioid users (Condon et al. 2022) and slightly lower than those of community members in a large epidemiological study (MacDonald et al. 2016). Participants in Profiles A and B (the groups with the overall best sleep health) reported the lowest levels, although these differences were not statistically significant after statistical adjustment. Reports of more childhood trauma among participants in Profiles C and D who had clinical insomnia suggest the contributions of childhood trauma to poor sleep health and are consistent with a large body of literature on childhood trauma and sleep [e.g.(Desch et al. 2023)]. For example, adverse childhood experiences, including physical and emotional abuse, neglect, parental incarceration, parental alcoholism, foster home placement, and community violence, had a dose-response relationship with insomnia symptoms into adolescence and young adulthood (Desch et al. 2023). Differences were found across the profiles on physical and mental health, with the best health among participants in profiles A and B, underscoring the importance of sleep to health.

Generally, participants scored above one on the Brief Addiction Monitoring use scale and below 12 on the protective factors scale (the respective cutoffs are ≥1 and ≤ 12), suggesting participants’ needs for further clinical attention (e.g. assessment for pharmaco-therapy, meeting with a case manager) (U.S. Department of Veterans Affairs 2009). Since participants in Profiles C and D (clinical insomnia and clinical insomnia with excessive daytime sleepiness, respectively) scored, on average, at or above 12 on the risk factor scale (the cutoff is ≥12), they may particularly benefit from more intensive treatment (e.g. relapse prevention skills training) (Burke et al. 2024). Given that all participants were early in MOUD treatment (i.e. 6 months or less), this may be an opportune time to focus on improvements in insomnia. The odds ratios with significant 95% confidence intervals show that these variables were consistently poorest in Profile D (clinical insomnia and excessive daytime sleepiness), the group with the poorest sleep and the worst scores on opioid use and risk factors, and best in Profile A, and the differences between Profile A and B and D were statistically significant after adjustment for other variables. However, it is unclear whether substance use (including polysubstance use as measured in the BAM risk scale) may be a cause or a consequence of poor sleep health (e.g. self-medication to improve sleep) or whether excessive daytime sleepiness may be a result of poor sleep or substance use. Treating sleep problems may also improve addiction behavior and vice versa. However, there have been few, if any, randomized controlled trials of the efficacy of treatment of sleep health in improving opioid use or vice versa. (Huhn and Finan 2022)

Levels of intersectional day-to-day discrimination, a variable that indicates multiple sources of discrimination, were lowest in Profile A and overall consistent with levels reported among sexual and gender minority groups and Black and Indigenous respondents (Scheim and Bauer 2019). This finding, in contrast with the lack of difference across the profiles in stigma specific to OUD, suggests that discrimination occurs due to multiple factors (e.g. gender, income, race, lifestyle) and contributes to sleep health. This is consistent with our previous finding within a sub-sample of individuals in the current study who participated in qualitative interviews that those who reported more discrimination and negative emotions from stigma reported more rumination that interfered with sleep (Nwanaji-Enwerem et al. 2024).

Some family, neighborhood, and social/community-level variables were associated with sleep profiles, and the directions of these relationships were consistent with our hypotheses. However, not all were statistically significant, especially after adjustment. Our finding that participants in Profiles C and D who had the worst insomnia symptoms reported less satisfaction with general family functioning may reflect the adverse effects of stress from less supportive family interactions, as in other groups with OUD (Hosseinbor et al. 2012). OUD contributes to impaired interactions or relationships, communication, and instrumental support, which may contribute to more worry, rumination, and difficulty falling or staying asleep. Our finding that the most positive perceptions of support from the community occurred in persons with better sleep health indicates the importance of social support to sleep health, as noted in other populations (Nam et al. 2018; Krause and Rainville 2020). Interventions to promote improved family functioning and strengthen community support are essential in addressing OUD (Kumar et al. 2021), as emphasized in a recent study of women on MOUD’s perspectives on insomnia treatment (Eglovitch et al. 2023) and practice guidelines (United States Substance Abuse and Mental Health Services Administration 2021), and they may also improve sleep.

The unadjusted associations between better perceived esthetic neighborhood quality, living in one’s own home, and more positive sleep health suggest that positive home and neighborhood attributes may be protective. However, the absence of an association between sleep health and social cohesion contrasts with our finding of an association with support from friends and a recent study among Black Americans in the same state, in which social cohesion was associated with better sleep quality (Nam et al. 2018). Although the absence of associations between the other neighborhood variables and sleep concurs with Nam et al. (2018), scores on neighborhood violence were higher in our sample than in previous studies (Mujahid et al. 2007; Nam et al. 2018). Yet, the lack of an association between neighborhood safety and violence and the sleep profiles contrasts with recent research that suggests the importance of neighborhood violence to sleep quality during childhood and across the lifespan (Poindexter et al. 2023; Saelee et al. 2023; Gaston et al. 2024; Saelee et al. 2024). In addition, the reason for better social cohesion in group D vs. group A in analyses controlling for other variables is contradictory to our expectations. Given that the difference was significant after controlling for living in one’s home, this may be a function of specific home environments. The reasons for the discrepancies between the current data and previous work are not known. While there may be no relationship between sleep and the selected environmental factors among patients on MOUD, the study may have been underpowered to detect differences across sleep profiles. Future studies should be prospectively powered to address meaningful differences and include objective neighborhood measures using objective graphical information systems or other methods.

The associations between multilevel factors and sleep health found in this study expand understanding beyond standard approaches that emphasize individual clinical and demographic characteristics and isolated sleep characteristics, such as sleep quality or duration alone. Our preliminary descriptions of four sleep profiles suggest that about 30% of the sample had reasonably good sleep, and the remaining groups differed on sleep duration, timing, and satisfaction, as indicated by the PSQI and PROMIS measures of sleep quality and the severity of insomnia symptoms. However, the causal nature of these must be examined in future studies. Understanding risk and protective multi-level factors may help identify adults on MOUD with differing profiles of sleep health and who may benefit from different sleep treatments (e.g. sleep extension, more regular timing, or cognitive behavioral therapy for insomnia). Addressing specific risks and bolstering protective factors through individual (e.g. trauma and other mental health concerns), family (e.g. family support), neighborhood, and social/community interventions (e.g. improved housing and safe neighborhoods) along with individual sleep intervention may improve sleep health among people on MOUD.

The strengths of this study include the sample of individuals early in MOUD, who are an underrepresented group in the sleep and addictions literature. Additional strengths include the focus on sleep health profiles, well-characterized self-report measures, and a conceptually-driven and well-detailed approach to the social-ecological model. The study was limited by self-report sleep measures and a cross-sectional design, which hindered causal or longitudinal attributions. Although the prevalence of sleep disordered breathing in the current sample is not known, it may be as high as 91% among opioid users with pain (Mubashir et al. 2020), and this may have influenced the findings. It was impossible to determine the number of sleep health phenotypes a priori. Therefore, we may not have had sufficient power to detect statistically significant differences across the profiles and due to the exploratory nature of this study, we used both adjusted and unadjusted p-values. Therefore, our interpretations are exploratory and merit further research.

This cross-sectional study comprised baseline data from a more extensive longitudinal study of the effects of sleep on relapse and retention of MOUD. These studies are essential to support family, neighborhood, and social interventions with traditional medical treatments (e.g. MOUD and cognitive-behavioral therapy) to promote sleep health. Our findings provide crucial foundational information to support future development and testing of interventions with individuals on MOUD.

## Figures and Tables

**Table 1.** Demographic characteristics of the sample (*N* = 165).

		Mean	SD
Age		42.0	11.4
		*N*	%
Sex	Male	92	55.8
	Female	73	44.2
Race	White	124	75.2
	Black /African American	16	9.7
	More than one race	21	12.7
	Unknown	4	2.4
Ethnicity	Hispanic or Latino	30	18.2
	Not Hispanic or Latino	134	81.2
	Unknown	1	0.6
Living Situation	Own home/apartment	126	76.4
	Assisted living	2	1.2
	Nursing home/skilled care facility	0	0
	Homeless	18	10.9
	Other	19	11.5
Living with	Alone	31	18.9
	With spouse or partner	65	39.6
	With family other than spouse	43	26.2
	With other	25	15.2
Education	Highschool or less	110	66.7
	Advanced course after high school	42	25.4
	Bachelor or higher	13	7.9
Employment	Full time (35+ hrs.)	66	33.0
	Part time (reg. hrs.)	19	9.5
	Part time (irreg. hrs.)	19	9.5
	Student	2	1.0
	Retired/disability	24	12.0
	Unemployed	69	34.5
	In controlled environment	1	0.5

**Table 2.** Individual, health, family, neighborhood, and community level characteristics.

Variables		Mean	SD	Cronbach’s Alpha
*Individual Levels*				
CTQ		47.5	21.3	0.95
BOSS – Agree		10.4	4.6	0.82
BOSS – Aware		16.4	3.9	0.77
(BOSS – Harm		8.0	4.5	0.83
Intersectional Day-to-day Discrimination Index		4.9	5.2	0.90
Income		$984.2	$3901.9	–
No. of days using more than one substance		8.3	10.7	–
% of 7 days using any Opioid		18.9	32.2	–
% of 7 days using Fentanyl/Opioid Use		34.2	38.8	–
Charlson Comorbidity Conditions		0.9	1.8	–
WHOQOL – Physical Health		13.6	3.2	0.74
WHOQOL – Psychological Health		13.9	3.1	0.80
BAM – Use		3.2	2.4	0.57
BAM – Risk Factors		10.5	5.0	0.65
BAM- Protective Factor		9.8	5.2	0.55
*Family Levels*				
Support from Partner/Family		18.8	4.1	0.83
Support from Outside the Home		26.5	7.4	0.89
McMaster Family Functioning		24.4	7.9	0.91
*Neighborhood Levels*	Range			
Esthetic Quality	1–5	2.7	1.1	0.85
Walking Environment	1–4.4	2.3	0.8	0.78
Availability of Healthy Foods	1–5	2.8	1.2	0.92
Safety Score	1–5	2.6	1.2	0.89
Violence Score	1–4	3.2	0.9	0.87
Social Cohesion Score	1–5	2.5	0.9	0.87
*Social Community Levels*				
Support from Friends	8–32	22.9	4.8	0.80
Support from Community	17–52	34.2	7.1	0.83
No. of High contact Roles	0–10	4.1	1.9	–
No. of People in Social Network	0–46	10.1	7.9	–
No. of Embedded Network	0–5	0.9	1.1	–
WHOQOL – Social Relationship	4–20	14.0	3.9	0.70
WHOQOL – Environment	4–20	14.1	3.1	0.81

Note. Childhood Trauma Questionnaire- CTQ; Brief Opioid Stigma Scales- BOSS; World Health Organization Quality of Life- WHOQOL; Brief Addiction Monitor- BAM.

**Table 3.** Principal components analysis: regression coefficients for the sleep health variables.

	Component 1 (Insomnia & Sleep Quality)	Component 2 (Sleepiness & Sleep Duration)	Component 3 (Sleep Timing)
Insomnia (ISI)	**0.806**	−0.130	0.031
Sleepiness (ESS)	−0.019	−**0.669**	−0.100
Sleep Disturbance (PROMIS)	**0.801**	−0.176	0.080
PSQI Total	**0.882**	−0.024	0.031
Sleep Duration (PSQI): Hrs.	−0.009	**0.819**	−0.101
Sleep Latency (PSQI): Mins.	**0.593**	−0.206	0.034
Sleep Efficiency (PSQI): %	−**0.896**	−0.205	0.038
Sleep Midpoint (PSQI)	−0.034	0.039	**0.993**

Note. Insomnia Severity Index- ISI; Epworth Sleepiness Scale- ESS; Sleep Disturbance Short Form- PROMIS; Pittsburgh Sleep Quality Index- PSQI.

**Table 4.** Sleep health profiles from the K-mean clustering (*N* = 165) and demographic characteristics of the profiles.

	Profile A Healthy Sleep *N* = 50 (30.3%)	Profile B Moderate Insomnia Symptoms with Late Sleep Timing *N* = 34 (20.6%)	Profile C Clinical Insomnia Symptoms w Long Sleep Duration *N* = 42 (25.5%)	Profile D Clinical Insomnia w Excessive daytime Sleepiness *N* = 39 (23.6%)
Demographics	Mean (SD)/N (%)	Mean (SD)/N (%)	Mean (SD)/N (%)	Mean (SD)/N (%)
Age	43.7 (11.0)	38.9 (11.8)	42.2 (10.7)	42.4 (11.9)
Male	29 (58.0%)	22 (64.7%)	17 (40.5%)	24 (61.5%)
Non-Hispanic White	36 (72.0%)	19 (55.9%)	31 (73.8%)	28 (71.8%)
Living Own Home	39 (78.0%)	31 (91.2%)	31 (73.8%)	25 (64.1%)
Living Alone	10 (20.0%)	5 (15.1%)	5 (11.9%)	28 (28.2%)
High School or Less	31 (62.0%)	21 (61.8%)	29 (69.1%)	29 (74.4%)
Sleep Outcomes	Mean (SD)	Mean (SD)	Mean (SD)	Mean (SD)
Insomnia (ISI)	6.38 (5.10)	11.79 (6.41)	14.79 (4.81)	15.95 (5.35)
Sleepiness (ESS)	5.98 (4.61)	7.68 (4.78)	5.55 (3.52)	12.49 (5.86)
Sleep Disturbance (PROMIS)	46.96 (8.95)	56.80 (9.10)	61.08 (5.96)	63.64 (7.88)
PSQI Total	5.74 (2.76)	9.38 (3.78)	12.60 (3.30)	12.21 (2.98)
Sleep Latency (PSQI): Mins.	20.64 (17.86)	41.32 (29.21)	48.95 (35.51)	76.08 (74.04)
Sleep Duration (PSQI): Hrs.	8.09 hr (1.56)	6.79 hr (1.59)	8.64 hr (1.68)	5.38 hr (1.63)
Sleep Efficiency (PSQI): %	82.1% (10.5)	74.1% (17.5)	57.6% (13.6)	67.3% (19.6)
Sleep Midpoint (PSQI)	2:11 AM (1.14)	5:35 AM (1.14)	3:04 AM (1.34)	2:41 AM (1.08)

Note. Insomnia Severity Index- ISI; Epworth Sleepiness Scale- ESS; Sleep Disturbance Short Form- PROMIS; Pittsburgh Sleep Quality Index- PSQI.

**Table 5.** Comparisons of individual, family, neighborhood, and social community levels across sleep health profiles.

Variables	Profile A Healthy Sleep N = 50 Mean (SD)	Profile B Moderate Insomnia/late sleep timing N = 34 Mean (SD)	Profile C Clinical Insomnia/long sleep duration) N = 42 Mean (SD)	Profile D Clinical Insomnia/excess sleepiness N = 39 Mean (SD)	*Unadj P-value	**Adj. P-value
*Individual Levels*						
CTQ	44.7 (19.6)	41.3 (18.5)	52.7 (23.7)	53.3 (22.7)	.0469	.1513
BOSS – Agree	10.7 (5.0)	8.6 (4.2)	11.4 (4.7)	10.8 (4.4)	.0739	.1128
BOSS – Aware	16.1 (4.4)	17.1 (3.5)	17.7 (2.5)	17.2 (3.5)	.2011	.2075
BOSS – Harm	8.2 (5.1)	7.2 (3.8)	8.6 (4.7)	8.5 (5.1)	.6280	.6739
Intersectional Day-to-day Discrimination	3.3 (3.9)	5.0 (5.7)	6.2 (5.8)	5.8 (5.6)	.0566	.0498
Income	656 (1449)	980 (3588)	405 (1001)	917 (4229)	.8321	.7910
No. of days using more than one substance	6.2 (9.6)	11.5 (11.7)	6.6 (9.7)	9.56 (11.5)	.0976	.0532
% of days using Opioid	13.6 (27.0)	24.4 (37.5)	25.6 (37.5)	12.9 (28.2)	.2120	.2243
% of days using Fentanyl/Opioid Use	31.5 (36.0)	49.3 (41.5)	36.6 (41.2)	27.3 (36.8)	.1290	.1223
Charlson Comorbidity Conditions	0.8 (1.1)	0.7 (1.6)	1.0 (1.5)	1.2 (2.6)	.6475	.5134
WHOQOL – Physical Health	15.0 (2.9)	14.0 (3.1)	12.5 (3.5)	11.8 (2.8)	.0001	.0002
WHOQOL – Psychological Health	14.7 (3.4)	14.1 (3.7)	13.4 (3.0)	12.8 (2.7)	.0480	.0955
BAM– Use	2.4 (2.2)	3.5 (1.7)	3.3 (2.5)	4.2 (3.2)	.0221	.0327
BAM – Risk Factors	7.9 (4.4)	11.0 (5.5)	12.0 (4.5)	13.5 (4.1)	<.0001	<.0001
BAM- Protective Factor	10.8 (5.3)	9.3 (3.9)	8.9 (4.9)	9.9 (5.7)	.3148	.1415
*Family Levels*						
Support from Partner/Family	19.4 (4.0)	18.8 (4.2)	18.7 (3.9)	18.1 (4.2)	.5429	.6423
Support from Outside the Home	28.4 (6.7)	26.7 (6.4)	24.7 (9.2)	25.8 (7.5)	.1362	.1511
McMaster Family Functioning	21.7 (7.5)	23.2 (7.3)	27.7 (8.8)	26.3 (8.7)	.0052	.0081
*Neighborhood Levels*						
Esthetic Quality	2.5 (1.1)	2.4 (1.1)	2.8 (1.1)	3.2 (1.1)	.0130	.0545
Walking Environment	2.3 (0.8)	2.1 (0.7)	2.5 (0.8)	2.4 (0.9)	.2599	.4415
Availability of Health Foods	2.7 (1.1)	2.6 (1.1)	3.0 (1.2)	3.1 (1.2)	.1823	.2931
Safety Score	2.5 (1.1)	2.3 (1.0)	2.7 (1.3)	2.8 (1.3)	.2679	.6675
Violence Score	3.3 (0.9)	3.3 (0.8)	3.2 (0.8)	2.9 (1.0)	.1777	.4721
Social Cohesion Score	2.3 (0.9)	2.5 (0.8)	2.6 (1.1)	2.6 (1.0)	.5312	.5505
*Social Community Levels*						
Support from Friends	23.7 (3.7)	23.0 (4.8)	24.2 (4.8)	21.9 (5.7)	.2085	.2440
Support from Community	37.1 (7.2)	34.9 (5.4)	33.0 (7.3)	31.7 (7.5)	.0070	.0112
No. of High contact Roles	4.5 (1.8)	4.2 (1.3)	3.6 (2.0)	3.8 (2.0)	.0916	.1230
No. of People in Social Network	12.1 (9.5)	9.7 (5.5)	8.7 (6.7)	8.6 (6.6)	.1227	.1414
No. of Embedded Network	1.1 (1.2)	0.9 (0.9)	0.8 (1.0)	0.8 (1.0)	.4964	.5166
WHOQOL – Social Relationship	15.2 (3.7)	13.7 (4.0)	13.6 (3.9)	13.0 (4.2)	.0622	.0743
WHOQOL - Environment	15.0 (3.2)	14.6 (3.0)	13.3 (3.4)	13.2 (2.9)	.0277	.0693

Note 1. Unadj. P-values were obtained from multinomial logistic regression to predict the sleep health profiles with each predictor.

** Adj. P-values were obtained from multinomial logistic regression with adjustment of living home status (Own home vs. others).

Note 2. Childhood Trauma Questionnaire- CTQ; Brief Opioid Stigma Scales- BOSS; World Health Organization Quality of Life- WHOQOL; Brief Addiction Monitor- BAM.

**Table 6.** Odds ratios of the sleep health profiles using multiple multinomial logistic regression.

	Profile B vs. A	Profile C vs. A	Profile D vs. A	
Variables	Adj. OR (95% CI)	Adj. OR (95% CI)	Adj. OR (95% CI)	*P*-value
*Model with Individual Level*				
WHOQOL – Physical Health	1.12 (0.60, 2.09)	0.61 (0.34, 1.07)	0.57 (0.31, 1.03)	.0765
BAM – Risk Factors	2.68 (1.39, 5.16)	2.17 (1.17, 4.00)	2.85 (1.48, 5.51)	.0066
Living in Own Home	6.05 (1.14, 30.86)	1.07 (0.37, 3.11)	0.71 (0.24, 2.09)	.0749

Note 1. Adj. OR is the adjusted odds ratio for Profile B, C, or D compared to Profile A estimated from the parsimonious model.

Note 2. World Health Organization Quality of Life- WHOQOL; Brief Addiction Monitor- BAM.

## References

[R1] Bastien CH, Valliéres A, Morin CM. 2001. Validation of the Insomnia Severity Index as an outcome measure for insomnia research. Sleep Med. 2(4):297–307. doi: 10.1016/s1389-9457(00)00065-4. 11438246

[R2] Bernstein DP, Fink L, Handelsman L, Foote J, Lovejoy M, Wenzel K, Sapareto E, Ruggiero J. 1994. Initial reliability and validity of a new retrospective measure of child abuse and neglect. American Journal of Psychiatry. 151(8):1132–1136. doi: 10.1176/ajp.151.8.1132. 8037246

[R3] Berro LF, Zamarripa CA, Talley JT, Freeman KB, Rowlett JK. 2022. Effects of methadone, buprenorphine, and naltrexone on actigraphy-based sleep-like parameters in male rhesus monkeys. Addict Behav. 135:107433. doi: 10.1016/j.addbeh.2022.107433. 35901553 PMC9495253

[R4] Bronfenbrenner U 1979. The ecology of human development: experiments by nature and design. Harvard University Press.

[R5] Brown BS, O’Grady KE, Battjes RJ, Katz EC. 2004. The Community Assessment Inventory–client views of supports to drug abuse treatment. J Subst Abuse Treat. 27(3):241–251. doi: 10.1016/j.jsat.2004.08.002. 15501377

[R6] Burke B, Clear B, Rollston RL, Miller EN, Weiner SG. 2024. An assessment of the one-month effectiveness of telehealth treatment for opioid use disorder using the brief addiction monitor. Subst Use Addctn J. 45(1):16–23. doi: 10.1177/29767342231212790. 38258856

[R7] Buysse DJ. 2014. Sleep health: can we define it? Does it matter? Sleep. 37(1):9–17. doi: 10.5665/sleep.3298. 24470692 PMC3902880

[R8] Buysse DJ, Reynolds CF 3rd, Monk TH, Berman SR, Kupfer DJ. 1989. The Pittsburgh Sleep Quality Index: a new instrument for psychiatric practice and research. Psychiatry Res. 28(2):193–213. doi: 10.1016/0165-1781(89)90047-4. 2748771

[R9] Cacciola JS, Alterman AI, Dephilippis D, Drapkin ML, Valadez C Jr., Fala NC, Oslin D, McKay JR. 2013. Development and initial evaluation of the Brief Addiction Monitor (BAM). J Subst Abuse Treat. 44(3):256–263. doi: 10.1016/j.jsat.2012.07.013. 22898042 PMC3602977

[R10] Cambron C, Gouripeddi R, Facelli JC. 2023. Healthcare provider reports on social determinants of health in opioid treatment. Psych. 5(1):60–69. https://www.mdpi.com/2624-8611/5/1/7.

[R11] Charlson ME, Pompei P, Ales KL, MacKenzie CR. 1987. A new method of classifying prognostic comorbidity in longitudinal studies: development and validation. J Chronic Dis. 40(5):373–383. doi: 10.1016/0021-9681(87)90171-8. 3558716

[R12] Chrobok AI, Krause D, Winter C, Plörer D, Martin G, Koller G, Adorjan K, Canolli M, Adam R, Wagner EM, 2020. Sleeping patterns in patients with opioid use disorder: effects of opioid maintenance treatment and detoxification. J Psychoactive Drugs. 52(3):203–210. doi: 10.1080/02791072.2020.1751900. 32299305

[R13] Cohen S, Doyle WJ, Skoner DP, Rabin BS, Gwaltney JM Jr.. 1997. Social ties and susceptibility to the common cold. JAMA. 277(24):1940–1944. https://www.ncbi.nlm.nih.gov/pubmed/9200634. doi: 10.1001/jama.1997.03540480040036. 9200634

[R14] Condon EM, Londono Tobon A, Mayes LC, Sadler LS. 2020. Acceptability and feasibility of hair and salivary biomarker collection among multiethnic school-age children. Matern Child Health J. 24(7):865–874. doi: 10.1007/s10995-020-02926-2. 32356128 PMC7378972

[R15] Condon EM, Tobon AL, Holland ML, Slade A, Mayes L, Sadler LS. 2022. Examining Mothers’ Childhood Maltreatment History, Parental Reflective Functioning, and the Long-Term Effects of the Minding the Baby^®^ Home Visiting Intervention. Child Maltreat. 27(3):378–388. doi: 10.1177/1077559521999097. 33678048 PMC13430977

[R16] Desch J, Bakour C, Mansuri F, Tran D, Schwartz S. 2023. The association between adverse childhood experiences and insomnia symptoms from adolescence to adulthood: evidence from the Add Health study. Sleep Health. 9(5): 646–653. doi: 10.1016/j.sleh.2023.06.001. 37419708

[R17] Eglovitch M, Parlier-Ahmad AB, Legge C, Chithranjan S, Kolli S, Violante S, Dzierzewski JM, Huhn AS, Wilkerson A, Martin CE. 2023. Patient-reported preferences for sleep interventions among women receiving buprenorphine for opioid use disorder. Front Psychiatry. 14: 1244156. doi: 10.3389/fpsyt.2023.1244156. 37779614 PMC10537926

[R18] Finlay M, Erwin JA, Skeiky L, Hansen DA, Layton ME, Quock R, Van Dongen HPA, Wilson M. 2023. Nighttime sleep and respiratory disturbances in individuals receiving methadone to treat opioid use disorder. J Addict Nurs. 34(4):E180–E188. doi: 10.1097/JAN.0000000000000470. 37772999

[R19] Gaston SA, Alhasan DM, Johnson DA, Hale L, Harmon QE, Baird DD, Jackson CL. 2024. Perceived childhood neighborhood safety and sleep health during childhood and adulthood among a cohort of African American women. Sleep Med. 117:115–122. doi: 10.1016/j.sleep.2024.03.004. 38531166 PMC12311802

[R20] Grandner MA, Jackson NJ, Izci-Balserak B, Gallagher RA, Murray-Bachmann R, Williams NJ, Patel NP, Jean-Louis G. 2015. Social and behavioral determinants of perceived insufficient sleep. Front Neurol. 6:112. doi: 10.3389/fneur.2015.00112. 26097464 PMC4456880

[R21] Hamilton E, Carr A. 2016. Systematic review of self-report family assessment measures. Fam Process. 55(1):16–30. doi: 10.1111/famp.12200. 26582601

[R22] Hernandez E, Griggs S. 2024. Sleep health among adults in outpatient opioid use disorder treatment: a systematic review. J Psychosoc Nurs Ment Health Serv. 62(1):19–26. doi: 10.3928/02793695-20230622-04. PMC10761602 37379124

[R23] Hosseinbor M, Bakhshani NM, Shakiba M. 2012. Family functioning of addicted and non-addicted individuals: a comparative study. Int J High Risk Behav Addict. 1(3): 109–114. doi: 10.5812/ijhrba.7514. 24971246 PMC4070117

[R24] Huhn AS, Finan PH. 2022. Sleep disturbance as a therapeutic target to improve opioid use disorder treatment. Exp Clin Psychopharmacol. 30(6):1024–1035. doi: 10.1037/pha0000477. 34110889 PMC8660927

[R25] Jalali MS, Botticelli M, Hwang RC, Koh HK, McHugh RK. 2020a. The opioid crisis: a contextual, social-ecological framework. Health Research Policy and Systems. 18(1): 87. doi: 10.1186/s12961-020-00596-8. 32762700 PMC7409444

[R26] Jalali MS, Botticelli M, Hwang RC, Koh HK, McHugh RK. 2020b. The opioid crisis: need for systems science research. Health Res Policy Syst. 18(1):88. doi: 10.1186/s12961-020-00598-6. 32771004 PMC7414582

[R27] Jean-Louis G, Grandner MA, Seixas AA. 2022. Social determinants and health disparities affecting sleep. Lancet Neurol. 21(10):864–865. doi: 10.1016/S1474-4422(22)00347-7. 36115351

[R28] Johns MW. 1991. A new method for measuring daytime sleepiness: the Epworth sleepiness scale. Sleep. 14(6):540–545. doi: 10.1093/sleep/14.6.540. 1798888

[R29] Jones CM, Han B, Baldwin GT, Einstein EB, Compton WM. 2023. Use of medication for opioid use disorder among adults with past-year opioid use disorder in the US, 2021. JAMA Netw Open. 6(8):e2327488–e2327488. doi: 10.1001/jamanetworkopen.2023.27488. 37548979 PMC10407686

[R30] Kelly SM, O’Grady KE, Schwartz RP, Peterson JA, Wilson ME, Brown BS. 2010. The relationship of social support to treatment entry and engagement: the Community Assessment Inventory. Subst Abus. 31(1):43–52. doi: 10.1080/08897070903442640. 20391269 PMC2856126

[R31] Kim B, Troxel WM, Dubowitz T, Hunter GP, Ghosh-Dastidar B, Chaix B, Rudolph KE, Morrison CN, Branas CC, Duncan DT. 2023. Neighborhood built environment and sleep health: a longitudinal study in low-income and predominantly African-American neighborhoods. Am J Epidemiol. 192(5):736–747. doi: 10.1093/aje/kwad016. 36691683 PMC10423630

[R32] Krause N, Rainville G. 2020. Exploring the relationship between social support and sleep. Health Educ Behav. 47(1):153–161. doi: 10.1177/1090198119871331. 31452389

[R33] Kumar N, Oles W, Howell BA, Janmohamed K, Lee ST, Funaro MC, O’Connor PG, Alexander M. 2021. The role of social network support in treatment outcomes for medication for opioid use disorder: a systematic review. J Subst Abuse Treat. 127:108367. doi: 10.1016/j.jsat.2021.108367. 34134871 PMC9022048

[R34] Lin C, Cousins SJ, Zhu Y, Clingan SE, Mooney LJ, Kan E, Wu F, Hser YI. 2024. A scoping review of social determinants of health’s impact on substance use disorders over the life course. J Subst Use Addict Treat. 166:209484. doi: 10.1016/j.josat.2024.209484. 39153733 PMC11418584

[R35] MacDonald K, Thomas ML, Sciolla AF, Schneider B, Pappas K, Bleijenberg G, Bohus M, Bekh B, Carpenter L, Carr A, 2016. Minimization of childhood maltreatment is common and consequential: results from a large, multinational sample using the childhood trauma questionnaire. PLoS One. 11(1):e0146058. doi: 10.1371/journal.pone.0146058. 26815788 PMC4729672

[R36] Mansfield AK, Keitner GI, Dealy J. 2015. The family assessment device: an update. Fam Process. 54(1):82–93. doi: 10.1111/famp.12080. 24920469

[R37] McCurry MK, Avery-Desmarais S, Schuler M, Tyo M, Viveiros J, Kauranen B. 2023. Perceived stigma, barriers, and facilitators experienced by members of the opioid use disorder community when seeking healthcare. J Nurs Scholarsh. 55(3):701–710. doi: 10.1111/jnu.12837. 36317787

[R38] Morin CM, Belleville G, Bélanger L, Ivers H. 2011. The Insomnia Severity Index: psychometric indicators to detect insomnia cases and evaluate treatment response. Sleep. 34(5):601–608. doi: 10.1093/sleep/34.5.601. 21532953 PMC3079939

[R39] Mubashir T, Nagappa M, Esfahanian N, Botros J, Arif AA, Suen C, Wong J, Ryan CM, Chung F. 2020. Prevalence of sleep-disordered breathing in opioid users with chronic pain: a systematic review and meta-analysis. J Clin Sleep Med. 16(6):961–969. doi: 10.5664/jcsm.8392. 32105208 PMC7849655

[R40] Mujahid MS, Diez Roux AV, Morenoff JD, Raghunathan T. 2007. Assessing the measurement properties of neighborhood scales: from psychometrics to econometrics. Am J Epidemiol. 165(8):858–867. doi: 10.1093/aje/kwm040. 17329713

[R41] Nam S, Whittemore R, Jung S, Latkin C, Kershaw T, Redeker NS. 2018. Physical neighborhood and social environment, beliefs about sleep, sleep hygiene behaviors, and sleep quality among African Americans. Sleep Health. 4(3):258–264. doi: 10.1016/j.sleh.2018.03.002. 29776620 PMC5961740

[R42] National Institute on Minority Health & Health Disparities. 2017. NIMHD Research Framework. Retrieved June 15 from https://nimhd.nih.gov/researchFramework.

[R43] Nwanaji-Enwerem U, Beitel M, Oberleitner DE, Gaeta Gazzola M, Eggert KF, Oberleitner LMS, Jegede O, Zheng X, Redeker NS, Madden LM, 2024. Correlates of perceived discrimination related to substance use disorders among patients in methadone maintenance treatment. J Psychoactive Drugs. 56(4):530–540. doi: 10.1080/02791072.2023.2230571. 37399330 PMC10761588

[R44] Nwanaji-Enwerem U, Redeker NS, O’Connell M, Barry D, Iheanacho T, Knobf TM, Scheinost D, Wang K, Yaggi K, Sadler LS. 2024. Experiences of stigma and discrimination compounded by intersecting identities among individuals receiving medication for opioid use disorder. J Health Care Poor Underserved. 35(1):94–115. https://www.ncbi.nlm.nih.gov/pubmed/38661862. doi: 10.1353/hpu.2024.a919810. 38661862 PMC12908579

[R45] Nwanaji-Enwerem U, Sadler LS, O’Connell M, Barry D, Knobf TM, Jeon S, Scheinost D, Yaggi K, Redeker NS. 2024. “It’s all connected:” A mixed methods study of insomnia, stigma, and discrimination among individuals on medication for opioid use disorder. Sleep Health. 10(1):31–40. doi: 10.1016/j.sleh.2023.09.004. 37980246 PMC12964356

[R46] Pantell M, Rehkopf D, Jutte D, Syme SL, Balmes J, Adler N. 2013. Social isolation: a predictor of mortality comparable to traditional clinical risk factors. Am J Public Health. 103(11):2056–2062. doi: 10.2105/AJPH.2013.301261. 24028260 PMC3871270

[R47] Poindexter M, Stokes A, Mellman TA. 2023. Neighborhood stress predicts fear of sleep independently of posttraumatic stress disorder. Behav Sleep Med. 21(2):185–192. doi: 10.1080/15402002.2022.2067162. 35471154 PMC10292665

[R48] Robinson SM, Sobell LC, Sobell MB, Leo GI. 2014. Reliability of the Timeline Followback for cocaine, cannabis, and cigarette use. Psychol Addict Behav. 28(1):154–162. doi: 10.1037/a0030992. 23276315

[R49] Rosa EM, Tudge J. 2013. Urie Bronfenbrenner’s theory of human development: its evolution from ecology to bio-ecology [Peer Reviewed]. Journal of Family Theory & Review. 5(4):243–258. doi: 10.1111/jftr.12022.

[R50] Saelee R, April-Sanders AK, Bird HR, Canino GJ, Duarte CS, Lugo-Candelas C, Suglia SF. 2024. Self-reported neighborhood stressors and sleep quality among Puerto Rican young adults. Sleep Health. 10(3):295–301. doi: 10.1016/j.sleh.2024.01.008. 38570224 PMC11162948

[R51] Saelee R, Haardörfer R, Johnson DA, Gazmararian JA, Suglia SF. 2023. Racial/Ethnic and sex/gender differences in sleep duration trajectories from adolescence to adulthood in a US National Sample. Am J Epidemiol. 192(1): 51–61. doi: 10.1093/aje/kwac156. 36004702 PMC10144618

[R52] Scheim AI, Bauer GR. 2019. The Intersectional Discrimination Index: development and validation of measures of self-reported enacted and anticipated discrimination for intercategorical analysis. Soc Sci Med. 226:225–235. doi: 10.1016/j.socscimed.2018.12.016. 30674436

[R53] Skevington SM, Lotfy M, O’Connell KA, Group, W. 2004. The World Health Organization’s WHOQOL-BREF quality of life assessment: psychometric properties and results of the international field trial. A report from the WHOQOL group. Qual Life Res. 13(2):299–310. doi: 10.1023/B:QURE.0000018486.91360.00. 15085902

[R54] Sobell LC, Sobell MB, Leo GI, Cancilla A. 1988. Reliability of a timeline method: assessing normal drinkers’ reports of recent drinking and a comparative evaluation across several populations. Br J Addict. 83(4):393–402. doi: 10.1111/j.1360-0443.1988.tb00485.x. 3395719

[R55] United States Department of Veteran’s Affairs. 2009. Brief addiction monitor (BAM) with scoring & clinical guidelines. Retrieved June 27 from https://www.mentalhealth.va.gov/providers/sud/docs/.

[R56] United States Substance Abuse and Mental Health Services Administration. 2021. Medications for Opioid Use Disorder. Treatment Improvement Protocol (TIP) Series 63. (Publication No. PEP21-02-01-002.). Rockville, MD: Substance Abuse and Mental Health Services Administration.

[R57] Wilkerson AK, McRae-Clark AL. 2021. A review of sleep disturbance in adults prescribed medications for opioid use disorder: potential treatment targets for a highly prevalent, chronic problem. Sleep Med. 84:142–153. doi: 10.1016/j.sleep.2021.05.021. 34153796 PMC8503844

[R58] Yang LH, Grivel MM, Anderson B, Bailey GL, Opler M, Wong LY, Stein MD. 2019. A new brief opioid stigma scale to assess perceived public attitudes and internalized stigma: evidence for construct validity. J Subst Abuse Treat. 99:44–51. doi: 10.1016/j.jsat.2019.01.005. 30797393 PMC6716158

[R59] Yu L, Buysse DJ, Germain A, Moul DE, Stover A, Dodds NE, Johnston KL, Pilkonis PA. 2011. Development of short forms from the PROMIS sleep disturbance and sleep-related impairment item banks. Behav Sleep Med. 10(1):6–24. doi: 10.1080/15402002.2012.636266. 22250775 PMC3261577

